# Expression of Phosphofructokinase Is Not Sufficient to Enable Embden-Meyerhof-Parnas Glycolysis in *Zymomonas mobilis* ZM4

**DOI:** 10.3389/fmicb.2019.02270

**Published:** 2019-09-27

**Authors:** Magdalena M. Felczak, Tyler B. Jacobson, Wai Kit Ong, Daniel Amador-Noguez, Michaela A. TerAvest

**Affiliations:** ^1^Department of Biochemistry and Molecular Biology, Michigan State University, East Lansing, MI, United States; ^2^Department of Bacteriology, University of Wisconsin-Madison, Madison, WI, United States; ^3^Department of Chemical and Biological Engineering, University of Wisconsin-Madison, Madison, WI, United States

**Keywords:** *Zymomonas mobilis*, glycolysis, Embden-Meyerhof-Parnas pathway, Entner-Doudoroff pathway, metabolic engineering

## Abstract

*Zymomonas mobilis* is a bacterium that produces ethanol from glucose at up to 97% of theoretical efficiency on a carbon basis. One factor contributing to the high efficiency of ethanol production is that *Z. mobilis* has a low biomass yield. The low biomass yield may be caused partly by the low ATP yield of the Entner-Doudoroff (ED) glycolytic pathway used by *Z. mobilis*, which produces only one ATP per glucose consumed. To test the hypothesis that ATP yield limits biomass yield in *Z. mobilis,* we attempted to introduce the Embden-Meyerhof-Parnas (EMP) glycolytic pathway (with double the ATP yield) by expressing phosphofructokinase (Pfk I) from *Escherichia coli.* Expression of Pfk I caused growth inhibition and resulted in accumulation of mutations in the *pfkA* gene. Co-expression of additional EMP enzymes, fructose bisphosphate aldolase (Fba) and triose phosphate isomerase (Tpi), with Pfk I did not enable EMP flux, and resulted in production of glycerol as a side product. Further analysis indicated that heterologous reactions may have operated in the reverse direction because of native metabolite concentrations. This study reveals how the metabolomic context of a chassis organism influences the range of pathways that can be added by heterologous expression.

## Introduction

The α-proteobacterium *Zymomonas mobilis* ZM4 is a promising chassis organism for lignocellulosic biofuel production because it produces ethanol at a high carbon efficiency; up to ~97% of the theoretical yield (Rogers et al., 1979; Karsch et al., 1983). The high carbon efficiency is caused partially by a low biomass yield; *Z. mobilis* produces ~40% less biomass per gram of substrate consumed than *Saccharomyces* spp. grown in the same conditions (Belaïch and Senez, 1965; Rogers et al., 1982). A low biomass yield is beneficial for bioprocessing because it enhances carbon efficiency and reduces the amount of microbial biomass, which must be treated as waste. However, it is not clear what metabolic and genetic determinants contribute to low biomass yield.

**Figure 1:**
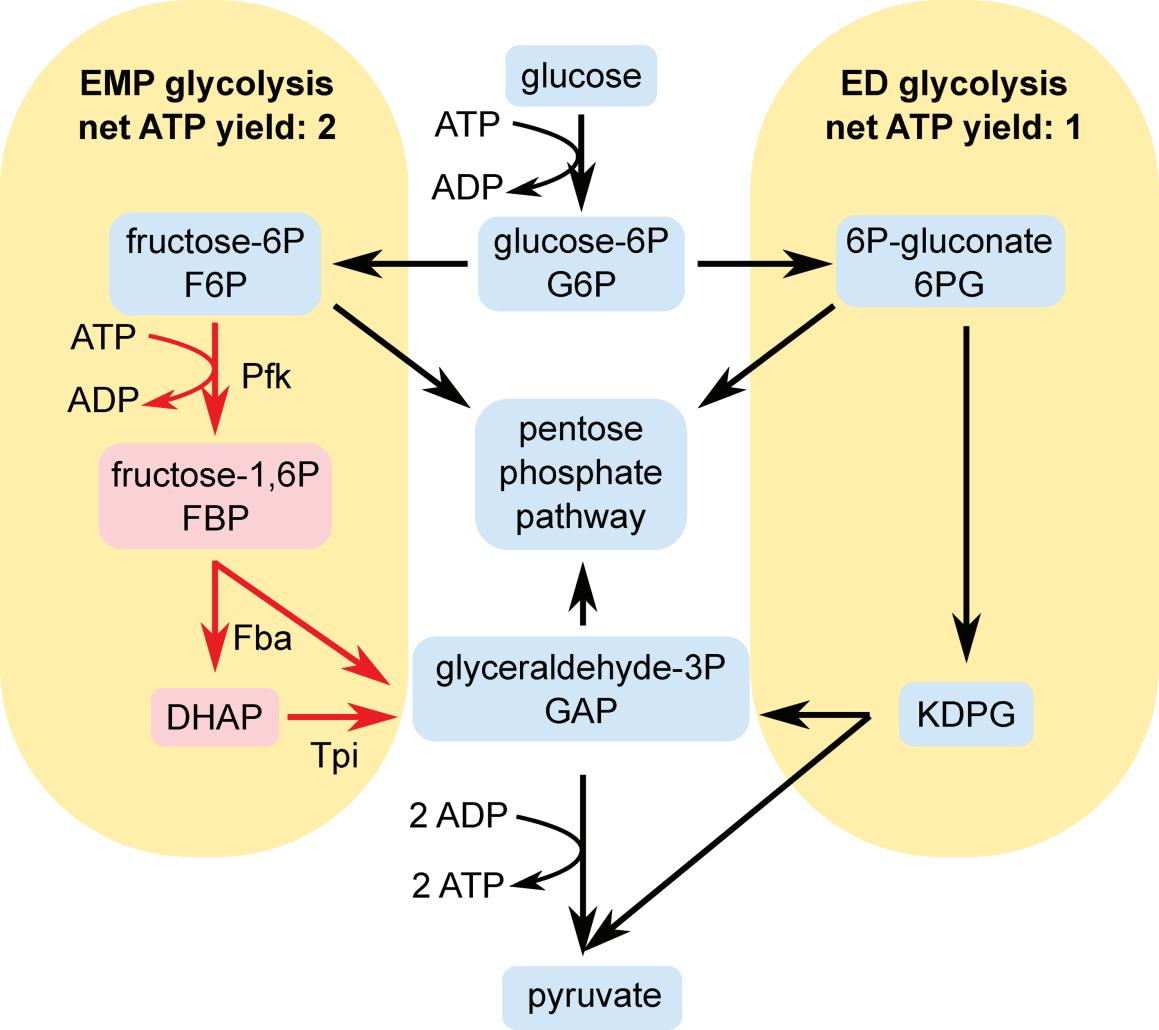
Simplified schematic of the EMP **(left)** and ED **(right)** pathways of glycolysis. Pfk, Fba, and Tpi reactions are indicated in red because heterologous expression of these enzymes was the major genetic change made in this study. Abbreviations: DHAP, dihydroxyacetone phosphate; KDPG, 2-keto-3-deoxy-6-phosphogluconate; Pfk, phosphofructokinase; Fba, fructose bisphosphate aldolase; Tpi, triosephosphate isomerase. The arrows show the directions of the flux through EMP or ED pathways and do not reflect reversibility/irreversibility of the reactions.

One factor leading to the low biomass yield of *Z. mobilis* may be its use of the Entner-Doudoroff (ED) pathway, which generates only one net ATP for each glucose molecule consumed (Figure 1). This pathway conserves less energy for growth and maintenance than the more commonly studied Embden-Meyerhof-Parnas (EMP) pathway, which generates a net of two ATP per glucose molecule consumed. The ED pathway is more commonly found in aerobes than anaerobes and *Z. mobilis* is unusual because it exclusively utilizes the ED pathway during anaerobic growth (Dawes et al., 1966; Conway, 1992). Aerobes may be better suited to tolerate the low ATP yield of the ED pathway because they also generate ATP *via* oxidative phosphorylation (Flamholz et al., 2013). Although *Z. mobilis* is a facultative anaerobe, it appears that oxidative phosphorylation is not an important ATP source in this organism (Bringer et al., 1984; Kalnenieks et al., 2008; Strazdina et al., 2012; Balodite et al., 2014; Jones-Burrage et al., 2019). Therefore, the ED pathway is likely the only major source of ATP in this organism, possibly contributing to its low biomass yield.

Previously, Chen et al. (2013) explored the possibility that the ED pathway limits ATP production and growth in *Z. mobilis* by attempting to complete an EMP glycolysis pathway. The authors determined by genome analysis and enzyme assays that phosphofructokinase is the only missing enzyme for EMP glycolysis in *Z. mobilis*, although fructose bisphosphate aldolase (Fba) and triose phosphate isomerase (Tpi) activities were low. To complete the pathway, they expressed a pyrophosphate (PP_i_)-dependent phosphofructokinase from a plasmid in *Z. mobilis*, alone or in combination with Fba and Tpi. Although ATP-dependent phosphofructokinase is more common, the authors chose a PP_i_-dependent version in an attempt to further enhance ATP yield of the engineered pathway. These modifications did not cause any significant change in growth. However, some shifts in metabolism were observed, including excretion of dihydroxyacetone (DHA). The authors concluded that an unknown bottleneck in lower glycolysis blocked processing of glyceraldehyde-3-phosphate (GAP).

In this study, we made a renewed effort to understand the connection between ATP yield and biomass yield in *Z. mobilis* by attempting to complete the EMP glycolysis pathway using an ATP-dependent phosphofructokinase. Similar to Chen et al. (2013), we found that introduction of EMP enzymes does not complete an EMP pathway. However, unlike PP_i_-dependent phosphofructokinase, expression of the ATP-dependent enzyme inhibited growth. We determined that homeostatic levels of glycolytic intermediates in *Z. mobilis* are incompatible with EMP flux, which explains why previous attempts to introduce EMP glycolysis have failed. As efforts to engineer *Z. mobilis* for biofuel production continue, it will be essential to include metabolomic data in the pathway design process to enhance the success rate of heterologous biosynthetic pathways.

## Materials and Methods

### Growth Media

Lysogeny broth (LB, Miller, Accumedia) was used to grow *E. coli* strains. ZRMG medium was used to grow *Z. mobilis* and consisted of 1% yeast extract, 2% glucose, and 15 mM KH_2_PO_4_. Solid ZRMG medium also contained 15 g agar per 1 L. Zymomonas recovery medium (ZRecM) consisted of 5 g yeast extract, 10 g tryptone, 0.25 g MgSO_4_, and 2.5 g (NH_4_)_2_SO_4_ in 1 L H_2_O. Spectinomycin was added to a final concentration of 100 μg/ml in cultures of *Z. mobilis* carrying pRL814 and derivative plasmids (50 μg/ml was used for *E. coli* cultures). IPTG (Goldbio) was added to desired final concentrations from 100 mM stock solutions. Dihydroxyacetone (DHA) was from Merck and glycerol was from Sigma-Aldrich.

### Bacterial Strains and Plasmids

*E. coli* strains used included Mach1 and MG1655. Wild-type *Zymomonas mobilis* subsp. *mobilis* ZM4 (ATCC 31821) and the plasmid pRL814 were obtained from Dr. Robert Landick. pRL814 contains a spectinomycin resistance gene (*aadA1*) and an IPTG-inducible superfolder *gfp* (T7A1-O34 promoter) and was described previously (Ghosh et al., 2019). Plasmids were transformed into *Z. mobilis* ZM4 variants with enhanced transformation efficiency PK15397 (ZM4Δ*mrr*) and PK15407 (ZM4Δ*mrr*Δ*hspS*Δ*cas3*) (P. Lal and P. Kiley, manuscript in preparation). PK15397 was used for the pRL*pfkA* construct, and PK15407 was used for all other constructs. Under the conditions used here, PK15407 and PK15397 displayed no observable differences in growth or metabolism compared with ZM4. pRL*pfkA* was synthesized by the Joint Genome Institute (JGI) by replacing the *gfp* gene of pRL814 with a synthetic version of the *pfkA* gene from *E. coli* MG1655. pRL*pfkA_tpiA_fbaA* bearing *pfkA*, *tpiA*, and *fbaA* genes from *E. coli* was constructed using NEBuilder High Fidelity DNA assembly kit (New England Biolabs). A three-piece assembly was used, with Hind III digested pRL*pfkA* as the backbone and PCR-amplified *tpiA* (b3919) and *fbaA* (b2925) as inserts. *tpiA* and *fbaA* were amplified from MG1655 genomic DNA using primers listed in Table 1. These primers introduced a FLAG-tag (GACTACAAAGACGATGACGACAAG) at the end of each insert. The pRL*pfkA_tpiA_fbaA* construct resulted in production of all three EMP enzymes in *E. coli* but only Pfk I with Tpi or Pfk I with Fba in *Z. mobilis* due to recombination (Supplementary Figure S2). The *pfkA_tpi* strain occasionally gained a fast-growth phenotype that may have been due to mutations, albeit infrequently (data not shown). Fast-growing colonies and cultures were not analyzed.

**Table 1:** Primers used for construction of pRL*pfkA_tpi_fbaA* and pRL*pfkA_tpi_fbaA*′ plasmids bearing identical or unique FLAG sequences, respectively.

Primer sequence	Primer use
5′AAAGACGATGACGACAAGTAAGCTTGCCATCTTCCTTTATTCGC 5′GTCATCGTCTTTGTAGTCAGCCTGTTTAGCCGCTTC 5′ACAGGCTGACTACAAAGACGATGACGACAAGTAAGCAACTTGAAGTATGACGAGTATAAG 5′AGGAATTCGATATCAAGCTTACTTGTCGTCATCGTCTTTGTAGTCCAGAACGTCGATCGC 5′ATCGTCATCCTTGTAGTCAGCCTGTTTAGCCGCTTC 5′ACAGGCTGACTACAAGGATGACGATGACAAATAAGCAACTTGAAGTATGACGAGTATAAG 5′AGGAATTCGATATCAAGCTTATTTATCATCATCATCTTTGTAATCCAGAACGTCGATCGC 5′CTTAGATTCAATTGTGAGCGG 5′CCTCGCTAACGGATTCACC	Tpi-FLAG forward Tpi-FLAG reverse Fba-FLAG forward Fba-FLAG reverse Tpi-NR FLAG reverse Fba-NR FLAG forward Fba-NR FLAG reverse Gfp-upstream fw Gfp-downstream rv

*Tpi or fbaA genes were amplified from E. coli MG1655 DNA and assembled with Hind III-digested pRLpfkA using NEBuilder High Fidelity DNA assembly kit. Gfp-upstream and Gfp-downstream primers, which anneal to the backbone of pRL814, were used to confirm the presence of inserts. Tpi-NR and Fba-NR FLAG; primers with nonredundant FLAG sequences*.

In a renewed attempt to co-express *pfkA*, *fbaA*, and *tpi* in ZM4, we constructed a vector containing all three genes with non-redundant FLAG sequences (pRL*pfkA_tpiA_fbaA*′) to minimize the chance of recombination. In this new attempt, the above FLAG tag sequence was replaced with GACTACAAGGATGACGATGACAAA for *tpi* and GATTACAAAGATGATGATGATAAA for *fbaA*. Assembly products were transformed into *E. coli* Mach1 and colony PCR was performed on spectinomycin-resistant transformants using primers specific to the pRL814 backbone, upstream and downstream of the cloning site to confirm correct assembly (Table 1). Plasmids were isolated using a plasmid extraction kit (Qiagen) and the sequence was confirmed by Sanger sequencing (Research Technology and Support Facility, Michigan State University). Expression of proteins was verified in whole cell lysates by Western blotting.

### Bacterial Growth

For growth kinetics performed using a plate reader (BioTek Synergy H1), starter cultures were inoculated from glycerol stocks in 5 ml of ZRMG with spectinomycin and grown anaerobically to OD 1.0–3.0. Different strains were inoculated from glycerol stocks at different times so that they reached the indicated OD_600_ at the same time. The starter cultures were diluted to OD_600_ = 0.1 with fresh ZRMG and wells of 96-well microtiter plates were filled with 150 μl of culture in triplicates. Cells were grown at 30°C with shaking for 50 h, and OD_600_ measurements were taken every 15 min. This experiment was performed inside an anaerobic chamber (Coy Laboratory Products). At the end of the experiment, triplicates were combined, OD_600_ was measured by a spectrophotometer with a 1-cm pathlength (Eppendorf BioPhotometer) for the biomass yield calculation, and 200 μl were saved for HPLC analysis. Doubling times were calculated from growth curves using the package “growthcurver” in R (Sprouffske and Wagner, 2016). Two-tailed *t*-tests were used to assess the statistical significance of differences in growth rate and biomass yield.

For large scale growth measurements, *Z. mobilis* strains were grown in 10 ml of indicated media, supplemented with spectinomycin. Cultures were grown in 15-ml culture tubes with caps, statically, in an anaerobic chamber at 30°C. Cultures were inoculated from glycerol stocks made from fresh transformants and grown until stationary phase (up to 110 h in the case of the *pfkA_tpi_fbaA* expressing strain). During growth OD_600_ was measured and samples were stored at −20°C for analysis by HPLC.

A conversion factor between OD_600_ and dry weight was determined by growing *Z. mobilis* in ZRMG anaerobically to OD_600_ = 2.0–3.0 in triplicate. A defined volume of each culture was filtered through pre-weighed, 22 μm nylon filter discs under vacuum. A total of five filters were used and total OD_600_ loaded onto each filter was calculated. Filters were dried in an oven at 105°C overnight. Filters were weighed after 5 h and after 20 h in the oven for stable weight. After subtracting the original weight of the filter, the dry weight per OD_600_ filtered was calculated. Values from five filters were averaged; the calculated conversion factor for 1 ml of culture was determined to be 0.215 ± 0.015 mg/OD.

### Electroporation Into *Z. mobilis*

Electrocompetent cells of Z. *mobilis*, were prepared as follows: 250 ml of ZRMG was inoculated from a single colony and grown overnight in a 250-ml flask, loosely covered at 30°C in oxic conditions and continued until OD_600_ = 0.4. Flasks were chilled on ice for over 30 min and cultures were spun at 4,000 ×*g* for 15 min. Pellets were resuspended in an equal volume of ice-cold Milli-Q H_2_O and left on ice for 10 min. Spinning was repeated and pellets resuspended in half of the initial volume of ice-cold Milli-Q H_2_O. After spinning as above, pellets were combined and resuspended in 10 ml of ice-cold 10% glycerol. After spinning at 4,000 ×*g* for 10 min, the pellet was resuspended in 300 μl of ice-cold 10% glycerol. Aliquots of electrocompetent cells were flash-frozen in liquid N_2_ and stored at −80°C, but freshly prepared cells were used for electroporation when possible. Electrocompetent cells (40 μl) were mixed with 1.0 μl of Type I restriction inhibitor (Lucigen) and 0.5–1 μg of plasmid DNA in 0.2 mm cuvettes and electroporated in a Micro Pulser (BioRad) set at EC-2. Cells were immediately resuspended in 1 ml of pre-warmed ZRecM and incubated at 30°C, for 5 h, aerobically. A 100 μl aliquot of the transformation or the entire transformation was spread on ZRMG plates supplemented with spectinomycin. Plates were protected with parafilm and incubated at 30°C for 2 days to 2 weeks until colonies appeared. All plates were incubated aerobically, except for those with *Z. mobilis* transformed with pRL*pfkA*_*tpi*_*fbaA*′.

### SDS-PAGE and Western Blotting

For protein expression, a portion of the glycerol stock was resuspended in a small volume of medium and identical volumes were used for inoculation of ZRMG to which IPTG was added to indicated concentrations. Cultures were grown aerobically for 16 h at 30°C. Samples were prepared as follows: a bacterial pellet was resuspended in lysis buffer (0.4 M Tris base, 30% glycerol, 1% SDS, 0.5% bromophenol Blue), boiled for 10 min, and vortexed to reduce viscosity. The equivalent of 1 ml of OD_600_ = 0.006 was loaded on 4–20% gradient Mini-PROTEAN TGX stain free gels (BioRad) and run in Tris/Glycine/SDS running buffer (BioRad) at 100 V until dye reached the bottom of the gel. All Blue Precision Plus protein standards (BioRad) were loaded alongside. Proteins were transferred to a nitrocellulose membrane in Trans-Blot-Turbo Transfer Buffer (BioRad) for 7 min in a Trans-Blot Turbo Transfer System (BioRad). Membranes were blocked with 3% BSA in TBST buffer (20 mM Tris-HCl pH 7.5, 150 mM NaCl, 0.01% Tween-20) for 1 h and incubated with monoclonal Anti-FLAG M2 antibody (Sigma-Aldrich) diluted to a final concentration of 1 μg/ml in 3% BSA in TBST overnight at 4°C. Membranes were washed three times in TBST for 2 h and incubated with 1:80,000 horseradish peroxidase-conjugated, rabbit anti-mouse antibody (Sigma-Aldrich), for 2 h at room temperature. Membranes were washed as above and chemiluminescent detection was performed with Clarity Western ECL (BioRad) for 5 min. Proteins were visualized using a Kodak Image Station 4000R, and net intensity of bands was measured by ROI analysis using Carestream Molecular Imaging Software.

### HPLC Analysis

Samples were prepared by centrifuging 0.2–1 ml of culture for 10 min at 13,000 rpm in a microcentrifuge to remove cells. The supernatant was transferred to a 2.0-ml glass HPLC vial with insert when needed (Vial: Restek, 21140; Cap: JG Finneran, 5395F09). Mixed standards of glucose and ethanol were prepared at concentrations of 5, 10, 20, 40, 60, 80, and 100 mM and 20, 40, 80, 160, 240, 320, and 400 mM, respectively. Glycerol and dihydroxyacetone (DHA, Merck) standards were made at 2, 4, 8, 16, 24, 32, and 40 mM. HPLC analysis was performed on a Shimadzu 20A HPLC, using an Aminex HPX-87H (BioRad) column with a Micro-guard Cation H^+^ guard column (BioRad) at 65°C. Compounds of interest were separated using a 0.6 ml/min flow rate, in 5 mM sulfuric acid with a 30-min run time and detected by refractive index and UV absorption at 271 nm (Shimadzu, RID-20A) maintained at 60°C. Samples were maintained at 10°C throughout analysis.

We used differences in RI and UV signal to distinguish between DHA and glycerol because both have similar retention times in this HPLC method. DHA shows a strong UV absorbance at 271 nm while glycerol does not (Supplementary Figure S4). During pilot experiments, we observed that some strains produced a mixture of glycerol and DHA during aerobic growth (Supplementary Figure S4, plate), but absence of UV_271_ signal at the DHA retention time in *pfkA_tpi_fbaA* cultures indicates that no DHA was produced during anaerobic growth (Supplementary Figure S4, large scale).

### LC/MS Analysis

Sample preparation: 10 ml of bacteria were grown in ZRMG supplemented with spectinomycin, to mid-logarithmic phase (OD_600_ = 0.4–0-5) in closed 15 ml culture tubes outside the anaerobic chamber to facilitate fast filtering and quenching of samples. The equivalent of 1 ml at OD_600_ 2.5 was collected on 0.22 μm nylon filter discs (Millipore) placed on a sintered glass funnel by pipetting bacterial culture onto the center of the filter under vacuum. Filters were immediately placed bacteria-down into 1.5 ml of −70°C methanol:acetonitrile:water (40:40:20) solvent to quench metabolism and extract metabolites and kept on dry-ice until all samples were collected. The entire bacterial suspension was transferred from the filters to Eppendorf tubes and spun at 4°C for 5 min at 16,000 ×*g*. Supernatant was transferred to clean Eppendorf tubes and dried immediately under N_2_ or stored at −80°C no longer than for 2 weeks, then dried before resuspending in 97:3 water:methanol with 10 mM tributylamine adjusted to pH = 8.2 by addition of ~10 mM acetic acid (Solvent A). Samples were analyzed by a HPLC-MS system consisting of a Vanquish UHPLC coupled *via* electrospray ionization (ESI) operated in negative mode to a Q Exactive orbitrap high-resolution mass spectrometer (Thermo Scientific). Liquid chromatography separation was conducted on an ACQUITY UHPLC BEH C_18_ column (1.7 μm particle size, 2.1 mm × 100 mm, Waters). Solvent A was as described above and solvent B consisted of 100% methanol. Total separation time was 25 min at 200 μl/min flow rate with the following gradient: 0 min, 5% B; 2.5 min, 5% B, 17 min, 95% B; 19.5 min, 95% B; 20 min, 5% B; and 25 min 5% B. Autosampler temperature was 4°C, and column temperature was 25°C. The mass spectrometry parameters were Full MS-SIM with 70–1,000 m/z, ACG target 1e6, maximum IT 40 ms, and resolution of 70,000. Compounds were identified by retention time and mass using MAVEN software. “Peak Area Top” values were normalized by OD equivalent of samples and used to generate heat maps of metabolites. Concentrations of selected metabolites were calculated from MS response factors of those metabolites. The *pfkA_fbaA* and *pfkA_tpi* strains were analyzed in a separate metabolomics experiment from *pfkA_tpi_fbaA* because the constructs were not all completed at the same time. However, identical growth conditions were used in both experiments, and strains were normalized to the pRL814 within each experiment.

## Results

### Expression of *E. coli pfkA* Causes a Growth Defect in *Z. mobilis*

**Figure 2:**
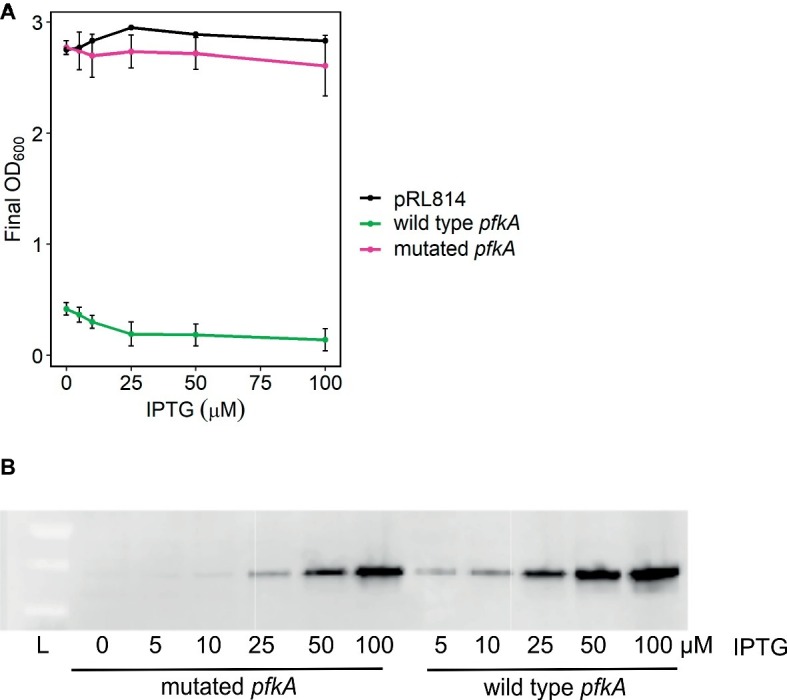
Growth of *Z. mobilis* bearing pRL*pfkA.* **(A)** Cultures were inoculated from glycerol stocks of slow or fast growing isolants of ZM4 bearing pRL*pfkA* and were grown overnight, aerobically, in rich medium with spectinomycin and indicated concentrations of IPTG. Average OD_600_ and standard deviations are from three biological replicates. **(B)** Whole cell lysates from overnight cultures in **(A)** were analyzed by Western blot. Equivalent of 1 ml at OD_600_ = 0.006 per lane was analyzed. Proteins were detected with anti-FLAG antibody. A representative blot is shown.

A control plasmid expressing *gfp* from an IPTG inducible promoter (pRL814) and a derivative plasmid with *E. coli pfkA* in place of *gfp* (pRL*pfkA*) were transformed into *Z. mobilis* (Supplementary Figure S1). *Z. mobilis* expressing *pfkA* grew poorly in rich medium: even with no IPTG added, cultures reached an OD_600_ < 0.5 after overnight growth versus an OD_600_ > 2.5 for *Z. mobilis* expressing *gfp*. The defect was observed without IPTG induction and worsened with increasing IPTG concentrations (“wild type *pfkA*” in Figure 2). However, after prolonged incubation (2–4 days), the growth defect abated in the majority of cultures and the strains began to show similar overnight growth to *Z. mobilis* with pRL814 (“mutated *pfkA*” in Figure 2). We observed that all cultures produced Pfk I protein in an IPTG-dependent manner (Figure 2B), but those that grew to a typical density for *Z. mobilis* had acquired mutations in *pfkA* that likely inactivated Pfk I. The locations of the mutations are included in Supplementary Table S1. Because of the rapid development of inactivating mutations and minimal growth of strains carrying wild-type *pfkA,* we were not able to study the effect of Pfk I on *Z. mobilis* directly. Therefore, we co-expressed additional EMP enzymes with Pfk I to overcome low expression levels of the native enzymes (Chen et al., 2013). This approach prevented the accumulation of mutations in the phosphofructokinase gene and allowed us to study phosphofructokinase activity in *Z. mobilis,* as described below.

### Co-expressing *tpi, fbaA,* or Both Stabilizes *pfkA*-Bearing Plasmids

We co-expressed *pfkA* with *tpi*, *fbaA*, or both to determine whether improved processing of the products of Pfk I would reduce toxicity. Enzyme assays performed by Chen et al. (2013) suggested low activity of these two enzymes. We generated three new constructs: pRL*pfkA_fbaA*, pRL*pfkA_tpi*, and pRL*pfkA_tpi_fbaA*. Initially, we constructed pRL*pfkA_tpi_fbaA,* which resulted in production of all three proteins in *E. coli* (Supplementary Figures S2A,B) but not in *Z. mobilis*. Instead, the pRL*pfkA_fbaA* and pRL*pfkA_tpi* constructs were obtained by spontaneous recombination between identical FLAG tags, which occurred after transformation of pRLp*fkA_tpi_fbaA* into *Z. mobilis* (Supplementary Figure S2C). We later learned that expression of all three enzymes in *Z. mobilis* slowed the growth of transformants significantly, which caused rare recombinant versions to appear on the plates first. A construct expressing all three proteins in *Z. mobilis* was obtained by replacing identical FLAG tag sequences in pRLp*fkA_tpi_fbaA* with unique ones, creating pRLp*fkA_tpi_fbaA′*, as described in the Section “Materials and Methods” and in Table 1. Changing the FLAG tags eliminated the background of recombined plasmids, and after 10–14 days of incubation, colonies with the correct construct were identified. Expression of the three heterologous proteins in ZM4 was confirmed by Western blot (Supplementary Figure S3). All three constructs were more stable than the original pRL*pfkA* construct in *Z. mobilis* (i.e., they did not accumulate inactivating mutations). We compared the growth of all strains expressing *pfkA* with different combinations of *tpi* and *fba* with ZM4 bearing pRL814.

**Figure 3:**
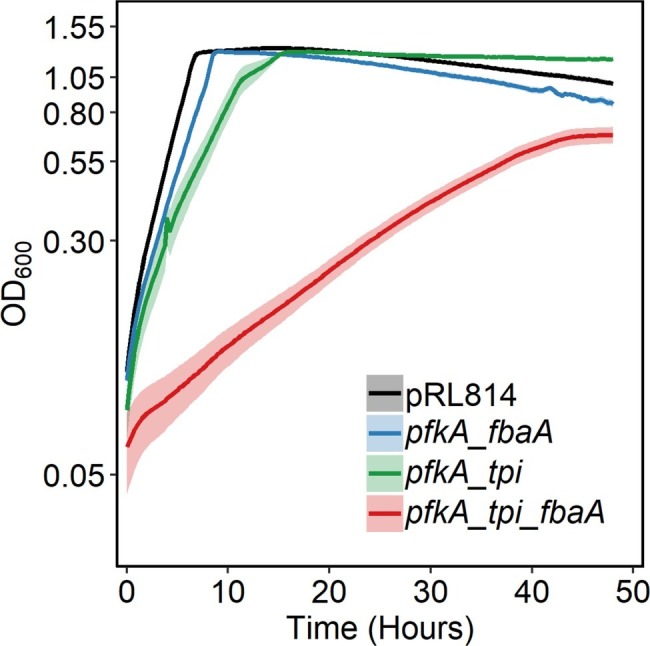
Growth of *Z. mobilis* expressing different EMP enzyme combinations in log_10_ scale. Strains were grown anaerobically in rich medium in 96-well microtiter plates (150 μl/well), at 30°C, with shaking. OD_600_ was measured every 15 min. Each growth curve is an average of at least three biological replicates with three technical replicates each. Error bars represent standard error. Note that some error bars are too small to be seen on this scale. The decrease in OD_600_ observed after 10 h of growth was caused by aggregation. To calculate biomass yield, the final OD_600_ of resuspended cells was measured by a spectrophotometer with a 1-cm pathlength (Table 2).

All *pfkA* bearing strains described above and the pRL814 control strain were grown without IPTG in a plate reader in anoxic conditions (Figure 3). All strains maintained wild-type EMP genes to the end of the experiment. We observed that the *pfkA_tpi_fbaA* strain grew slowly, with a doubling time of ~6 h compared to ~1 h for the pRL814 control (Table 2, *p* < 0.05). The biomass yield of the *pfkA_tpi_fbaA* strain was about half that of the pRL814 control (Table 2, *p* < 0.05). We also observed a reduced biomass yield in the *pfkA_tpi* strain, although the difference was not statistically significant. The biomass yield for the *pfkA_fbaA* strain was similar to the pRL814 control. The improved growth rate and yield of the *pfkA_fbaA* strain compared with the other *pfkA*-expressing strains suggests that improving fructose-1,6-bisphosphate (FBP) processing relieves some of the toxicity of phosphofructokinase activity. Surprisingly, adding the Tpi reaction to create the *pfkA_tpi_fbaA* strain negatively affected the growth rate and yield compared with the *pfkA_fbaA* strain.

**Table 2:** Doubling times, final OD_600_, and biomass yields of *Z. mobilis* strains expressing EMP enzymes.

Strain	Doubling time (min)	Final OD_600_	Biomass yield (g DW/mol glucose)
pRL814	56 ± 2	4.2 ± 0.1	8.6 ± 0.2
*pfkA_tpi*	101 ± 7	3.5 ± 0.6	7.2 ± 1.2
*pfkA_fba*	65 ± 4	4.2 ± 0.1	8.9 ± 0.2
*pfkA_tpi_fbaA*	385 ± 37	2.4 ± 0.4	5.1 ± 0.7

*Strains were grown on microtiter plates until stationary phase (50 h), as described in* Figure 3. *Doubling times were calculated from growth curves using the package “growthcurver” in R (Sprouffske and Wagner, 2016). Final OD_600_ of pooled triplicates of each culture were determined by spectrophotometer. Spent bacterial samples at time 0 and 50 h were analyzed by HPLC to determine glucose consumption. Biomass yield was calculated as increase in dry weight (DW) per glucose used. Each average and standard deviation is calculated from at least three biological replicates*.

### Expression of Fba With Pfk I Does Not Prevent Fructose-1,6-Bisphosphate Accumulation, While Adding Tpi Leads to Accumulation of DHAP

**Figure 4:**
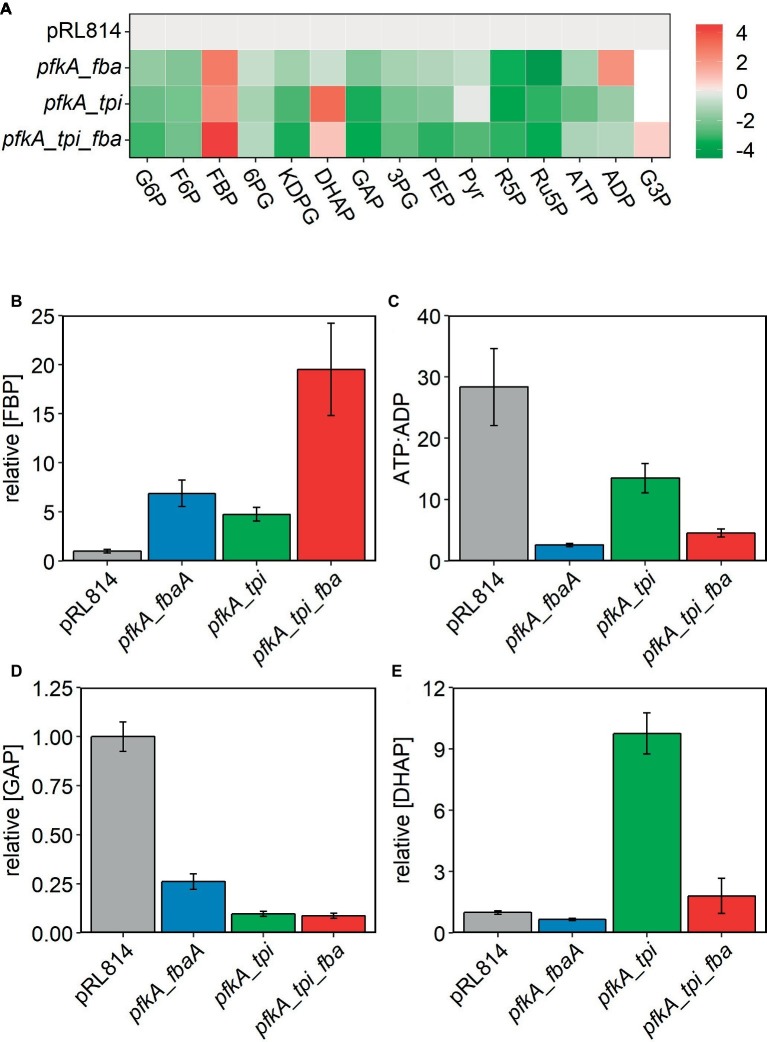
Relative abundance of glycolytic metabolites observed by LC/MS in *Z. mobilis* with pRL814 or constructs expressing glycolytic enzymes from *E. coli*. Cells were grown in rich medium to OD_600_ = 0.4–0.5 and metabolites were extracted and analyzed by LC/MS. Three biological replicates and two technical replicates were averaged for all strains except *pfkA_tpi*. Only one culture of *pfkA_tpi* showed characteristic slow growth at the time of harvest, and thus, only technical replicates of one culture were averaged. Data for the *pfkA_tpi_fbaA* strain are from a separate experiment, and signals were normalized to a pRL814 control for that experiment. Growth conditions were identical for both experiments. Only the experiment with *pfkA_tpi_fbaA* contains G3P observations. Six biological replicates were averaged for this strain. **(A)** Heatmap of intracellular metabolites shown as log_2_ fold change relative to pRL814. **(B–E)** Relative concentrations of metabolites to pRL814 control. Concentrations of metabolites were calculated from LC/MS signals and response factors of metabolite standards. Abbreviations: 6PG, 6-phosphogluconate; 3PG, 3-phosphoglycerate; PEP, phosphoenolpyruvate; R5P, ribose-5-phosphate; Ru5P, ribulose-5-phosphate; G3P, glycerol-3-phosphate and those in Figure 1.

We performed metabolomic analysis on *Z. mobilis* strains bearing plasmids pRL*pfkA_fbaA*, pRL*pfkA_tpi*, and pRL*pfkA*_*tpi_fbaA*′ to determine how expression of EMP enzymes affected the levels of glycolytic intermediates and provide insight into the observed growth differences. For all three strains, we observed that relative abundance of all glycolytic intermediates except for FBP and DHAP were lower than in the control strain. In the strain co-expressing Pfk I and Fba, FBP signal intensity was elevated ~6.9-fold compared with the pRL814 control, suggesting that FBP was not efficiently processed by heterologous Fba (Figure 4B). In the *pfkA_tpi* strain, FBP and DHAP increased by ~4.8- and ~9.8-fold, respectively (Figures 4A,B,E). The nearly 10-fold increase in DHAP even in the absence of Fba expression suggests that heterologous Tpi converts GAP produced by the ED pathway to DHAP (Figures 1, 4D,E). The DHAP to GAP conversion has a small *Δ_r_G*′*°*; therefore, the driving force and direction of the reaction (*Δ_r_G*′) are highly sensitive to substrate and product concentrations (Flamholz et al., 2012). The ATP to ADP ratio in all of the modified strains was also low compared to the pRL814 control (Figure 4C).

**Figure 5:**
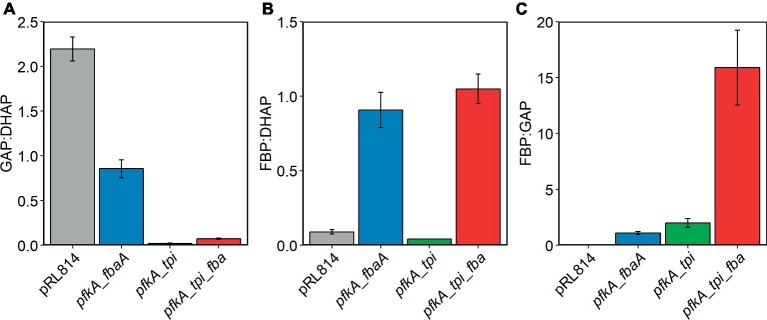
Relative concentrations of EMP metabolites in *Z. mobilis* ZM4 expressing EMP enzymes. Cells were grown and cellular metabolites were analyzed by LC/MS as described in the legend of Figure 4. Concentrations of metabolites were calculated from LC/MS signals and response factors of relevant metabolite standards. **(A)** GAP:DHAP, **(B)** FBP:DHAP, and **(C)** FBP:GAP.

In the strain expressing all three EMP enzymes, we observed accumulation of FBP to a level exceeding that in the *pfkA_fbaA* strain (~19.5-fold vs. pRL814). The *pfkA_tpi_fbaA* strain also accumulated DHAP, albeit to a lesser extent than the *pfkA_tpi* strain (~1.8-fold vs. pRL814). The GAP to DHAP ratio was 10–20 times lower than in the pRL814 control or any other strain expressing only the native Tpi (Figure 5A). There was also greater accumulation of FBP in the *pfkA_tpi_fbaA* strain than in the *pfkA_fbaA* strain. The increase in FBP accumulation caused by the addition of *tpi* suggests that increased DHAP production by reverse activity of Tpi may reduce the driving force for the Fba reaction or even reverse its direction (Figures 5B,C).

### Co-expression of *pfkA*, *tpi*, and *fbaA* Causes Glycerol Excretion at the Expense of Biomass Yield

**Figure 6:**
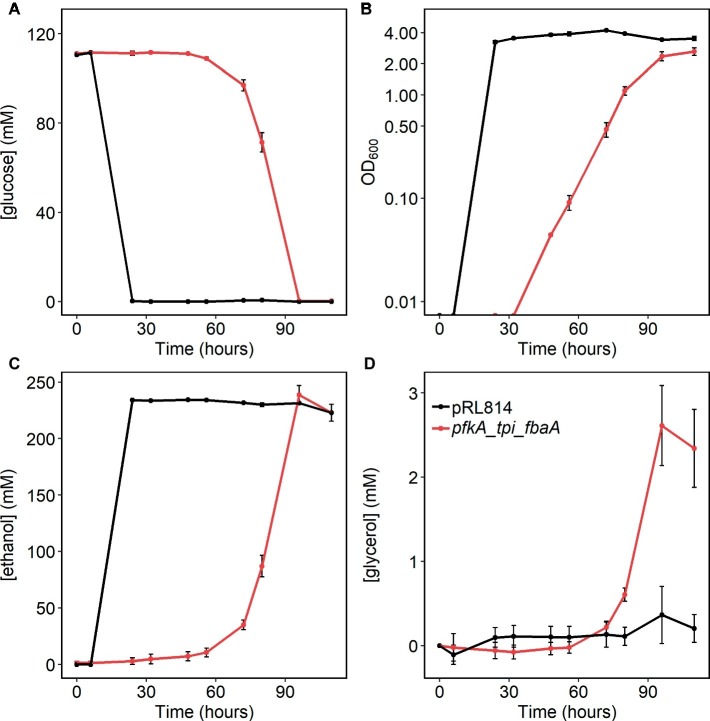
**(A)** Glucose consumption, **(B)** growth (log scale), **(C)** ethanol production, and **(D)** glycerol production in *Z. mobilis* expressing Pfk I, Fba, and Tpi vs. the pRL814 control during anaerobic growth. Cells were grown statically in rich medium at 30°C. Cultures were inoculated from glycerol stocks made from fresh transformants and grown until stationary phase (110 h). Graphs show average and standard deviations from three biological replicates of *Z. mobilis* with pRL814 or *Z. mobilis* with pRL*pfkA_tpi_fbaA.*

The *pfkA*_*tpi*_*fbaA* strain had a significantly reduced growth rate and biomass yield, along with altered metabolite concentrations; therefore, we also tested whether glucose uptake and ethanol production were affected in this strain. For this purpose, cells were grown anaerobically in rich medium and sampled for cell density and glucose and ethanol concentrations. We observed that growth, glucose consumption, and ethanol production were all slower in the *pfkA_tpi_fbaA* strain than in the pRL814 control, consistent with the previously calculated doubling time of 6 h (Figure 6). The final OD_600_ for the strain was significantly lower than that of the control strain and the calculated biomass yield was 5.1 ± 0.8 g DW/mol compared to 8.2 ± 0.2 g DW/mol for the control strain. Both strains consumed all available glucose by the end of the experiment and produced similar amounts of ethanol (Figures 6A,C).

Because Chen et al. (2013) reported DHA excretion by a strain expressing PP_i_-dependent Pfk with Tpi and Fba, we analyzed HPLC chromatograms for additional product peaks. We observed accumulation of glycerol in the *pfkA_tpi_fbaA* cultures (Figure 6D). Although glycerol and DHA elute at the same retention time in our HPLC protocol, we were able to determine their presence independently by their differing signals in refractive index versus UV absorbance detectors (Supplementary Figure S4). During pilot experiments, we observed that some strains produced a mixture of glycerol and DHA during aerobic growth, but no DHA was produced during anaerobic growth (Supplementary Figure S4). Therefore, we were able to quantify glycerol production in anaerobically grown cultures (see Section “Materials and Methods” for details). We calculated the molar ratio of glycerol produced per glucose used to be 0.02 ± 0.01 or ~1.2% of glucose carbon excreted to the medium as glycerol. Considering that wild-type *Z. mobilis* converts less than 3% of glucose carbon to biomass, excretion of ~1.2% of glucose carbon to the medium is consistent with the 50% lower biomass yield of the *pfkA_tpi_fbaA* strain compared with the pRL814 control. In the metabolomics analysis, we observed an increased signal for glycerol-3-phosphate (G3P) in the *pfkA_tpi_fbaA* strain compared to the pRL814 control (Figure 4A), suggesting that G3P dehydrogenase (ZMO1905) is the first step in converting DHAP to glycerol.

## Discussion

In contrast to our hypotheses, adding Pfk I activity to *Z. mobilis* did not increase the biomass yield or ATP to ADP ratio. Instead, it caused growth inhibition and elimination of Pfk I activity by accumulation of inactivating mutations. Previously, a PP_i_-dependent Pfk was expressed in *Z. mobilis* and did not cause growth inhibition (Chen et al., 2013). The PP_i_-dependent Pfk may have had a smaller impact on metabolism because PP_i_ hydrolysis likely provides less driving force than ATP hydrolysis. The standard free energy gain for ATP hydrolysis is greater than that for PP_i_ (−43.5 vs. −32.9 kJ/mol) and the ATP to ADP ratio in the cell is generally higher than the PP_i_ to P_i_ ratio (Chen et al., 2013). Therefore, strains expressing the PP_i_-dependent Pfk may have accumulated less FBP than the strains generated in our study. All strains with *pfkA* accumulated FBP intracellularly, indicating that carbon flux was blocked at the early steps of the EMP pathway. The blockage appeared to result in withdrawal of carbon from the ED and pentose phosphate pathways but without an alternative route for energy production. This metabolic disruption may have led to the apparent toxicity of Pfk I, which consumes ATP, leading to a reduced ATP to ADP ratio. It is also possible that FBP accumulation itself was toxic; FBP has been shown to inhibit phosphoenolpyruvate kinase in *S. aureus* (Zoraghi et al., 2010). A similar effect of FBP on *Z. mobilis* pyruvate kinase or inactivation of another enzyme cannot be excluded. Toxicity of DHAP and DHA to the cells may also be a reason for the observed decrease in growth rate of *tpi*-overexpressing strains (Bar-Even et al., 2012; Wang et al., 2018).

Although our results suggest that the reduction in growth rate results from EMP enzyme activities, it is possible that the metabolic burden caused by overexpression of heterologous enzymes also contributes. In a strain expressing only *pfkA,* this contribution seems to be minor, as *pfkA* expressing strains show growth inhibition only when active Pfk I is produced, while mutated versions of *pfkA* do not cause growth inhibition. In strains expressing *fbaA* or *tpi* together with *pfkA*, the metabolic burden should be similar or higher in the *pfkA_fba* strain (Fba is almost twice as large as Tpi) if based only on the level of protein expression. However, the *tpi*-expressing strain grows much slower than the *pfkA_fba*A strain, suggesting that activity is the dominant factor.

While we observed broad disruption of central metabolic pathways in *Z. mobilis* carrying EMP enzymes, our metabolomic analysis also points to a specific problem with EMP flux at the Tpi step. The accumulation of DHAP in the strain with *pfkA_tpi* suggests that GAP produced by the ED pathway was converted into DHAP, i.e., the reaction was proceeding in the reverse direction with respect to EMP glycolysis. The interconversion of GAP and DHAP operates close to equilibrium (*Δ_r_G*′*°* = +5.5 ± 1.1 kJ/mol for DHAP → GAP); therefore, the direction of the reaction is strongly influenced by the ratio of GAP to DHAP (Flamholz et al., 2012). In *E. coli*, this ratio is ~0.1, allowing the DHAP to GAP conversion to operate close to equilibrium (*Δ_r_G*′ = −0.2 ± 1.1 kJ/mol) (Park et al., 2016). However, in *Z. mobilis* with the control plasmid, we observed a GAP to DHAP ratio of >2.0, making the conversion of GAP to DHAP favorable (*Δ_r_G*′ = −7.2 ± 1.1 kJ/mol). Although introduction of pRL*pfkA_tpi_fbaA* reduced the GAP to DHAP ratio to a level similar to *E. coli*, it appears that DHAP to GAP conversion was still not efficient in this context. This incompatibility between the *Z. mobilis* metabolome and EMP flux may be due to a higher homeostatic GAP concentration in *Z. mobilis* than in *E. coli*. Jacobson et al. (2019) recently determined that the GAP concentration in *Z. mobilis* is 26 times higher than in *E. coli*. The high level of GAP likely results from the high cumulative free energy of the first reactions in the ED pathway (Flamholz et al., 2013). The high net flux of the ED pathway would affect the modified strains discussed here, because the native glycolytic route was still functional. It is also possible that the *Z. mobilis* GAP dehydrogenase may have a higher *K*_m_ than GAP dehydrogenases from EMP utilizing organisms and may require a higher concentration of GAP for the reaction to proceed efficiently. Currently, the *K*_m_ value for the *Z. mobilis* enzyme is not available.

Another reason why the GAP to DHAP ratio remained high compared with, e.g., *E. coli*, even when FbaA is expressed, is that accumulated DHAP is released by conversion to glycerol and excretion to the medium. This processing of DHAP results in an alternative fermentation pathway that does not contribute to energy conservation. The observed increase in cellular G3P in the *pfkA_tpi_fbaA* strain points to G3P dehydrogenase (NAD(P)^+^, ZMO1905) as the first enzyme in metabolizing DHAP. Glycerol-3-phosphatase would complete the pathway to produce glycerol. Both reactions leading to glycerol (DHAP → G3P and G3P → glycerol) have a negative free energy when substrate and product concentrations are equal, (*Δ_r_G*′*°* = −27.4 ± 1.6 kJ/mol and *Δ_r_G*′*°* = *−*10.8 ± 4.1 kJ/mol, respectively) providing an efficient way of reducing the excess of DHAP. Glycerol-3-phosphatase has not yet been annotated in the *Z. mobilis* genome thus far, so further research is necessary to clarify which pathways contribute to glycerol synthesis. Additional research is also necessary to elucidate the pathway for DHA production under aerobic conditions.

Proximity channeling could be another explanation for the difficulty in redirecting flux from one glycolytic pathway to another. This has been observed previously, where overexpression of homologous ED enzymes in *E. coli* did not capture significant flux from EMP pathway unless PTS was knocked out (Abernathy et al., 2019). Although there is no evidence of similar channels in *Z. mobilis,* it is possible that difficulties in redirecting glucose flux from ED pathway to EMP may be partially due to existence of ED proximity channels. However, decreases in abundances of ED and PPP metabolites (KDPG or Ru6P and R6P, respectively) support the model that G6P and F6P were redirected to the EMP pathway and converted to FBP. We cannot exclude that ED metabolites downstream of G6P are channeled, but these metabolites are not shared with EMP. Thus, proximity channeling in the ED pathway does not seem to be a likely reason for the lack of EMP flux in strains expressing EMP enzymes but further research should explore this possibility.

## Conclusion

In this study, we attempted to better understand the low biomass yield of *Z. mobilis*. We predicted that creation of a functional EMP pathway would reduce ethanol yield and increase biomass yield. In contrast to this prediction, we found that expression of the only missing EMP enzyme, Pfk I, was toxic to *Z. mobilis* ZM4. As a result, we were not able to determine whether use of the ED pathway is a determinant of the low biomass yield and rather studied *Z. mobilis* metabolism in more depth to determine why the EMP pathway did not operate as predicted. We found that adding phosphofructokinase alone did not create a functional EMP pathway. Adding two downstream enzymes to enhance FBP processing resulted in reversal of the Tpi reaction and excretion of excess DHAP as glycerol. The high native GAP to DHAP ratio combined with the capability of *Z. mobilis* to excrete glycerol suggests that widespread genetic modifications would be required to create a functional EMP pathway in this organism.

## Data Availability Statement

The datasets generated for this study are available on request to the corresponding author.

## Author Contributions

MF designed and performed the study and experiments, analyzed data, and drafted the manuscript. TJ performed metabolomics analysis, consulted on experimental design, and revised the manuscript. WO performed computational modeling that formed the basis of the study, and revised the manuscript. DA-N designed metabolomics portions of the study, consulted on data analysis, and revised the manuscript. MT designed the study and experiments, analyzed data, and drafted and revised the manuscript.

### Conflict of Interest

The authors declare that the research was conducted in the absence of any commercial or financial relationships that could be construed as a potential conflict of interest.
