# Supplementary material for: Expression of Phosphofructokinase Is Not Sufficient to Enable Embden-Meyerhof-Parnas Glycolysis in Zymomonas mobilis ZM4
Source: Front Microbiol. 2019 Sep 27;10:2270. doi: 10.3389/fmicb.2019.02270 (PMC6777484; doi:10.3389/fmicb.2019.02270)
Supplement: Supplementary file 1 [file Data_Sheet_1.docx]

**Supplementary Tables**

**Table S1**. Mutations in plasmid borne *pfkA* genes isolated from fast growing *Z. mobilis* transformants. Location of changed amino acids and possible defect in function was determined from the crystal structure of *E. coli* Pfk I (PDB accession number 1PFK (Shirakihara and Evans, 1988)).

| ***Isolate*** | ***Mutation*** | ***Change*** | ***Location within***  ***secondary structure*** | ***Location within***  ***tertiary structure*** | ***Possible defect*** |
| --- | --- | --- | --- | --- | --- |
| 2  11  14  16  17  18  19 | multiple  Δ536-554  G196A  ΔC43  G510A  Δ728-814  Δ375-407 | P15WG16RM17IN18T  Δ178-184  G66S  Frameshift  M170I  Δ261-271  Δ126-136 | Helix 1  Helix 7  Loop β-C α-3  Helix 1  β-F  Helix 11  Entire loop  β-E α-6 | Close to substrate site  Effector site  Unknown  Frameshift  Substrate site  Dimer interface  Substrate site | ATP/ADP binding  Effector binding  Unknown  Random polypeptide  F6P binding  Oligomerization  Catalysis |

**Supplementary Figures**

**
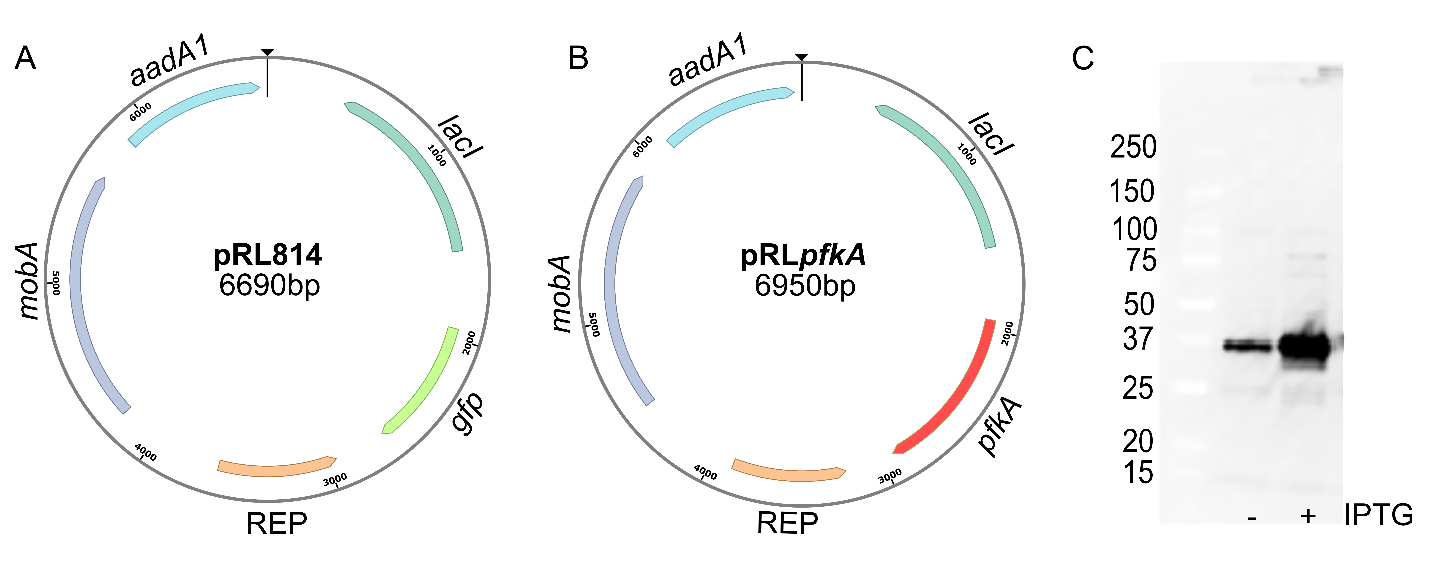
**

**Figure S1**. **A**. pRL814 expresses *gfp* from an IPTG inducible promoter, T7A1-O34 and contains spectinomycin resistance gene, *aadA1* (Ghosh et al., 2019). **B**. In pRL*pfkA,* the *gfp* gene was replaced with *E. coli* *pfkA* and a FLAG sequence was added to C-terminus. **C**. Expression of Pfk I-FLAG in *E. coli* Mach I*.*  Western blot of whole cell lysate with or without IPTG induction (100 µM). Equivalent of 0.006 OD_600_ was loaded on 4-20% gradient SDS-PAGE. Protein was detected using an anti-FLAG antibody. Numbers to the left indicate molecular weights of protein standards in kDa.


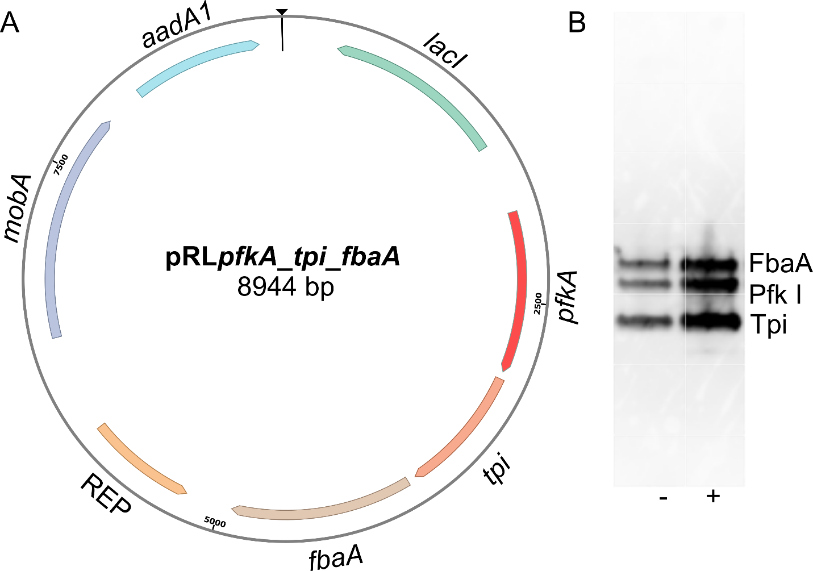


C

**Figure S2**. (**A**) In pRL*pfkA_tpi_fbaA,* *gfp* of pRL814 was replaced with *pfkA*, *tpi* and *fbaA* genes from *E. coli.* Each of the *E. coli* genes carried an identical FLAG-tag sequence at the C-terminus. (**B**) Expression of Pfk I, FbaA and Tpi genes in *E. coli* Mach I and (**C**) in *Z. mobilis* PK15407. Equivalent of 0.006 OD_600_ was loaded on 4-20% gradient SDS-PAGE. Protein was detected using an anti-FLAG antibody. Numbers to the left of blots show molecular weight of protein standards in kDa.


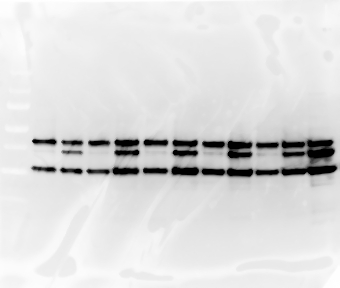


**- + - + - + - + IPTG**

**1 2 3 4 Isolate**

**50**

**37**

**25**

**Figure S3**. Expression of Pfk I, Tpi and Fba proteins bearing non-redundant FLAG sequences at in *Z. mobilis*. pRL*pfkA_tpi_fbaA’* bearing unique FLAG sequences at the C-terminus of each of the genes was introduced to ZM4 by electroporation. Spectinomycin-resistant transformants to ZM4 were used for inoculation of rich media w/o or with 100 µM IPTG and grown for 48 hrs, anaerobically, at 30^O^C. Equivalent of 0.006 OD_600_ of whole cell lysate was loaded on 4-20% gradient SDS-PAGE. Proteins were detected with anti-FLAG antibody. Numbers to the left of each blot show molecular weight of protein standards in kDa.


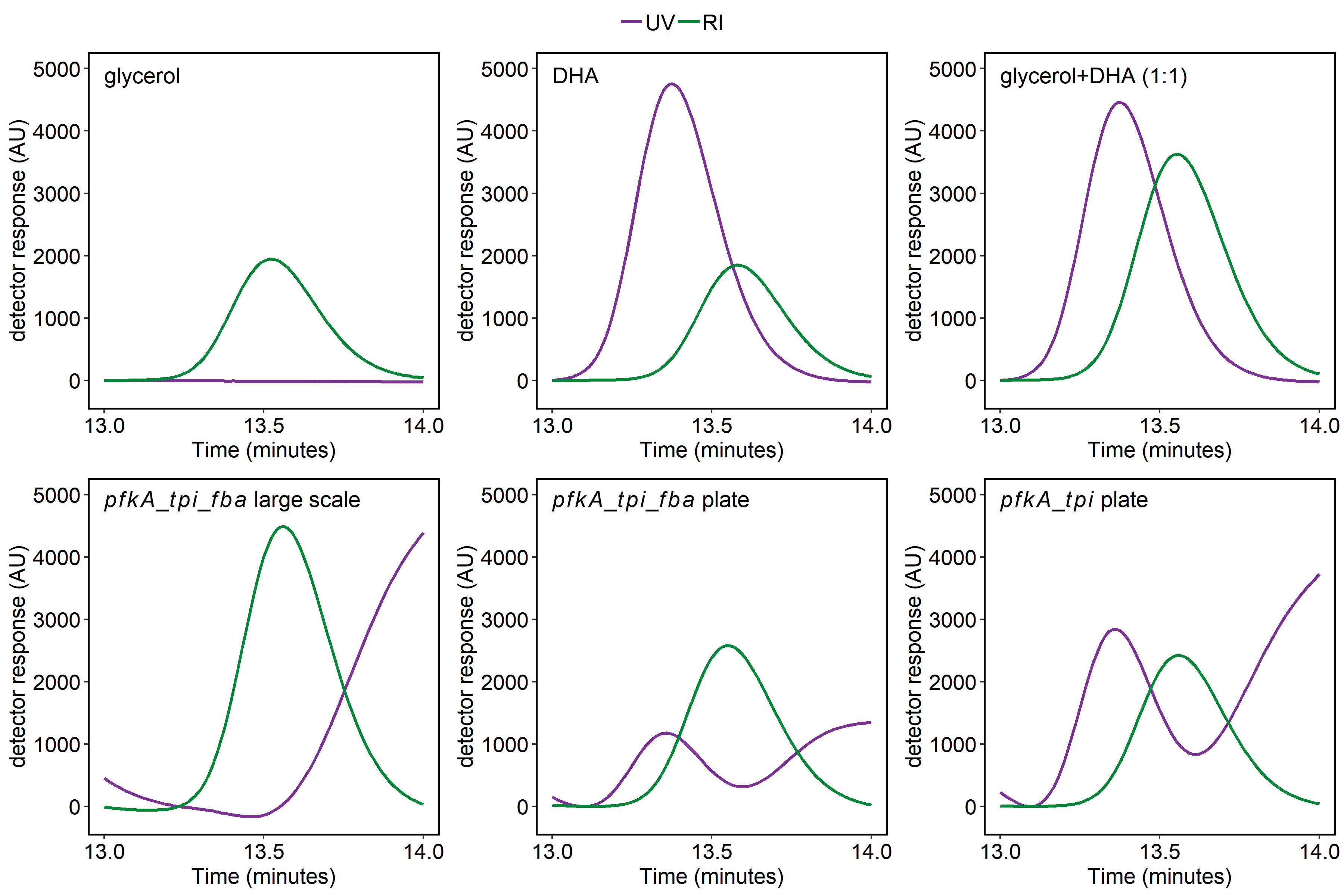


**Figure S4**. Chromatograms from HPLC analysis of supernatants from *pfkA_tpi_fbaA* and *pfkA tpi* cultures, collected by refractive index (RI) and UV detectors. Cells were grown in rich medium in 15 mL tubes (large scale) anaerobically or on 96-well microtiter plates, (plate), aerobically, to stationary phase. Culture supernatants or DHA and glycerol standards were run on an Aminex HPX-87H column and eluted with 5 mM H_2_SO_4_. RI, green; UV_271_, purple

**References**

Ghosh, I. N., Martien, J., Hebert, A. S., Zhang, Y., Coon, J. J., Amador-Noguez, D., et al. (2019). OptSSeq explores enzyme expression and function landscapes to maximize isobutanol production rate. *Metab. Eng.* 52, 324–340. doi:https://doi.org/10.1016/j.ymben.2018.12.008.

Shirakihara, Y., and Evans, P. R. (1988). Crystal structure of the complex of phosphofructokinase from *Escherichia coli* with its reaction products. *J. Mol. Biol.* 204, 973—994. doi:10.1016/0022-2836(88)90056-3.
